# Substance P Activates the Wnt Signal Transduction Pathway and Enhances the Differentiation of Mouse Preosteoblastic MC3T3-E1 Cells

**DOI:** 10.3390/ijms15046224

**Published:** 2014-04-11

**Authors:** Gang Mei, Zhenlv Zou, Su Fu, Liheng Xia, Jian Zhou, Yongtao Zhang, Yonghua Tuo, Zhao Wang, Dan Jin

**Affiliations:** 1 Department of Orthopaedics and Traumatology, Nanfang Hospital, Southern Medical University, 1838 North Guangzhou Avenue, Guangzhou 510515, Guangdong, China; E-Mails: meigang622@outlook.com (G.M.); qvbghdkk@163.com (Z.Z.); fusu321789@gmail.com (S.F.); smilefish_2009@163.com (L.X.); zhoujian321789@gmail.com (J.Z.); zhaoyan2016@gmail.com (Y.Z.); 2 Department of Orthopaedic, Wuzhou Red Cross Hospital, Wuzhou 543002, Guangxi, China; E-Mail: tyh2150@gmail.com; 3 School of Engineering and Materials Science, Queen Mary University of London, Mile End, London E1 4NS, UK; E-Mail: ctwz198412@googlemail.com

**Keywords:** substance P, Wnt signal transduction pathway, differentiation, MC3T3-E1 cells

## Abstract

Recent experiments have explored the impact of Wnt/β-catenin signaling and Substance P (SP) on the regulation of osteogenesis. However, the molecular regulatory mechanisms of SP on the formation of osteoblasts is still unknown. In this study, we investigated the impact of SP on the differentiation of MC3T3-E1 cells. The osteogenic effect of SP was observed at different SP concentrations (ranging from 10^−10^ to 10^−8^ M). To unravel the underlying mechanism, the MC3T3-E1 cells were treated with SP after the pretreatment by neurokinin-1 (NK1) antagonists and Dickkopf-1 (DKK1) and gene expression levels of Wnt/β-catenin signaling pathway components, as well as osteoblast differentiation markers (*collagen type I*, *alkaline phosphatase*, *osteocalcin*, and *Runx2*), were measured using quantitative polymerase chain reaction (PCR). Furthermore, protein levels of Wnt/β-catenin signaling pathway were detected using Western blotting and the effects of SP, NK1 antagonist, and DKK1 on β-catenin activation were investigated by immunofluorescence staining. Our data indicated that SP (10^−9^ to 10^−8^ M) significantly up-regulated the expressions of osteoblastic genes. SP (10^−8^ M) also elevated the mRNA level of c-myc, cyclin D1, and lymphocyte enhancer factor-1 (Lef1), as well as c-myc and β-catenin protein levels, but decreased the expression of *Tcf7* mRNA. Moreover, SP (10^−8^ M) promoted the transfer of β-catenin into nucleus. The effects of SP treatment were inhibited by the NK1 antagonist and DKK1. These findings suggest that SP may enhance differentiation of MC3T3-E1 cells via regulation of the Wnt/β-catenin signaling pathway.

## 1. Introduction

Neurons not only innervate skeletal muscles, but also function as a network innervating cancellous bone [1]. They contain a variety of peripherally released neurotransmitters, whose functional receptors have been found in bone cells [2,3]. Substance P (SP) is a neuropeptide present in primary sensory neurons and the central, as well as peripheral nervous systems [4–7]. SP is a member of the tachykinin family. This group of peptides consists of five subtypes: neuropeptide K, neurokinin A, neurokinin B, neuropeptide C, and SP. Thus far, three receptor subtypes have been identified for that group of peptides (NK-1, -2, and -3 receptor) [8]. SP is a mediator in many physiological and pathological processes. Recent studies have revealed function of SP in neurokinin-1 receptor (NK1-Rs) dependent modulation of osteoblastic bone formation [9]. Furthermore, SP is associated with bone metabolism, including osteoblastic bone formation and osteoclastic bone absorption. SP stimulates the proliferation and differentiation of bone marrow stromal stem cells [4,10–12].

Stability and localization of β-catenin is crucial for signal transduction in the Wnt/β-catenin pathway [13,14]. Canonical Wnts bind to the 7-transmembrane domain-spanned frizzled (Fz) receptor and low-density lipoprotein 5 and 6 (LRP5/6) co-receptors. This complex recruits the scaffolding protein Dishevelled (Dvl) and initiates a cascade of events. Dvl interacts with the destruction complex consisting of the scaffold protein Axin, adenomatous polyposis coli (APC) and glycogen synthase kinase-3 (GSK-3) [15,16]. Disruption of this complex leads to phosphorylation of GSK-3β and stabilization of β-catenin through dephosphorylation. Subsequent translocation of β-catenin into the nucleus leads to interaction with Tcf/Lef transcription factors and up-regulation of Wnt-responsive genes, including cyclin D1, c-jun, c-myc, and fra-1 [17–19]. Wnt signaling is tightly regulated by secreted regulatory proteins, such as Dickkopf (Dkk), which antagonizes LRP5/6 [20]. Over the past few years the Wnt/β-catenin-signaling pathway has been shown to be an important component of bone mass accrual, regulation, and maintenance [21–23]. However, the link between SP and the canonical Wnt/β-catenin signaling pathway in the case of osteogenic process has not been clarified.

Recent reports showed that calcitonin gene-related peptide, another neurotransmitter released by peripheral nerve system, promoted the differentiation of MC3T3-E1 osteoblast-like cells [24]. However, the effect of SP on the differentiation of pre-osteoblastic cell line and the related mechanism are still unknown. We hypothesized that SP may stimulate the differentiation of MC3T3-E1 cells via regulation of Wnt/β-catenin signaling pathway. In this study, the mice pre-osteoblastic cell line MC3T3-E1 was used as an *in vitro* model with osteogenic properties and fast growth. Our findings revealed that SP enhanced the differentiation of MC3T3-E1 cells and activated the Wnt/β-catenin signaling pathway.

## 2. Results and Discussion

### 2.1. Results

#### 2.1.1. Isolation, Screening, Alkaline Phosphatase (ALP) and Alizarin Red Staining of MC3T3-E1 Cells

**Figure 1.**
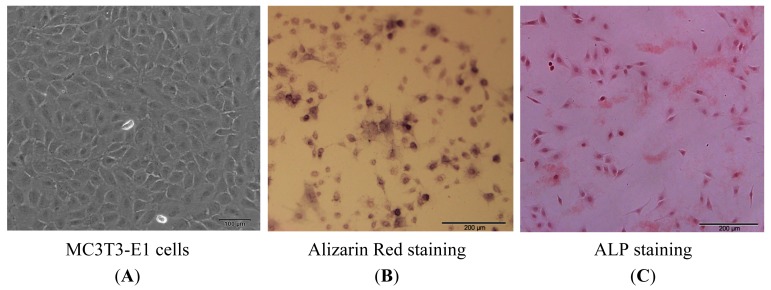
Growth and morphologic characteristics of MC3T3-E1 cells. (**A**) Cells grew in triangular and polygonal shapes; (**B**) Alizarin red staining for Ca deposits, extracellular matrix Ca deposits for matrix mineralization was measured using Alizarin red dye which bound with Ca; and (**C**) staining for alkaline phosphatase (ALP), cells were cultured in 6-well plates and incubated in AS-MX phosphate solution as substrate with Fast Red salt as stain, with matrix ALP activity shown as red stain.

To show the osteoblastic features of the MC3T3-E1 line, cells were used for alkaline phosphatase (ALP) and Alizarin red staining before exposure to the differentiation medium. ALP activity and presence of Ca deposits in extracellular matrix indicate that the MC3T3-E1 cells possess osteogenic properties (Figure 1).

#### 2.1.2. Influence of Substance P (SP) at Different Concentrations on Differentiation of the MC3T3-E1 Cells into Osteoblasts

**Figure 2.**
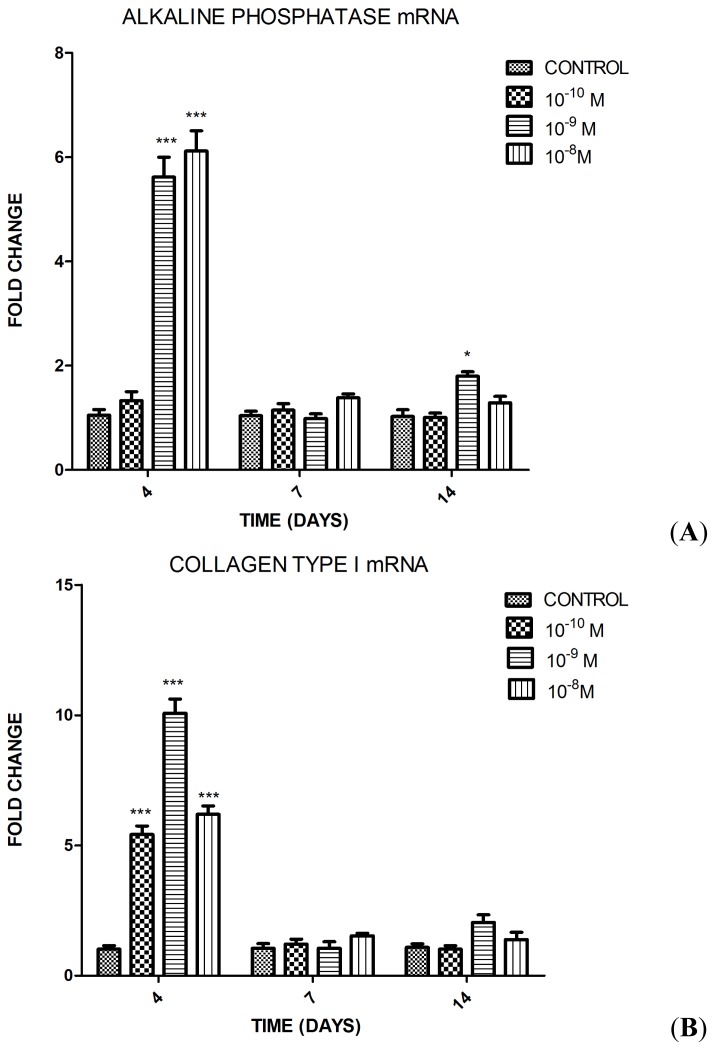

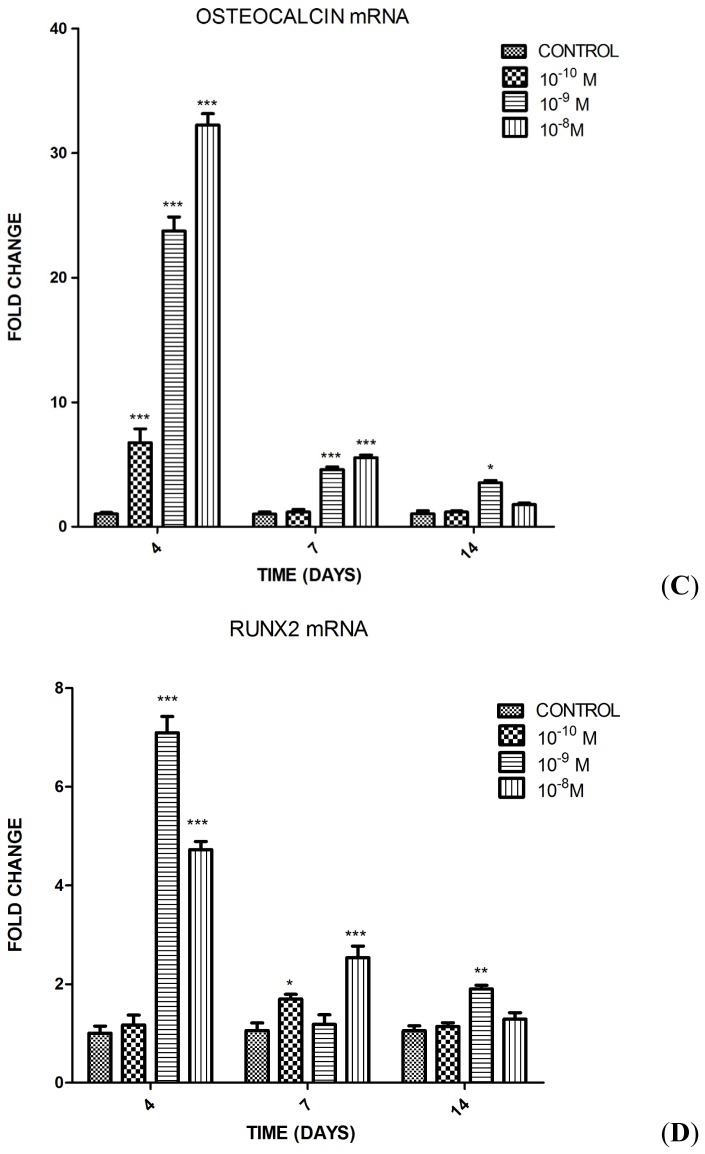
Effects of substance P (SP) treatment at days 4, 7, and 14 (10^−8^, 10^−9^ and 10^−10^ M) on gene expressions in MC3T3-E1 cells measured by qPCR for (**A**) alkaline phosphatase; (**B**) collagen type I; (**C**) osteocalcin; and (**D**) *Runx2* mRNA levels. The values reported are the mean ± SEM of three independent experiments. * *p* < 0.05 *versus* Control; ** *p* < 0.01 *versus* Control; and *** *p* < 0.001 *versus* Control.

The impact of SP at different concentrations on the differentiation of MC3T3-E1 cells into osteoblasts was assessed by testing the osteoblastic gene expressions (*collagen type I*, *alkaline phosphatase*, *osteocalcin*, and *Runx2*) using quantitative polymerase chain reaction (qPCR). Collagen type I and alkaline phosphatase were selected as early markers of osteoblastic differentiation. Osteocalcin was selected as a late marker of that process. Furthermore, the expression of *Runx2*, a transcription factor crucial in osteoblast differentiation, was examined. Three different concentrations of SP (10^−8^, 10^−9^, and 10^−10^ M) were tested in MC3T3-E1 cells at days 4, 7, and 14 in the osteoinductive medium. SP (10^−8^ M) significantly increased the level of alkaline phosphatase and collagen type I mRNA at day 4, significantly increased the levels of osteocalcin and *Runx2* mRNA at days 4 and 7. SP at concentration of 10^−9^ M, significantly increased the levels of alkaline phosphatase and *Runx2* mRNA at days 4 and 14, significantly increased the level of osteocalcin mRNA at days 4, 7, and 14 and collagen type I mRNA at day 4. Treatment with 10^−10^ M SP resulted only in elevated collagen type I and osteocalcin mRNA level at day 4 compared with the control group (group 4) (Figure 2).

#### 2.1.3. Impact of SP, Neurokinin-1 (NK1) Antagonist and Dickkopf-1 (DKK1) on the Differentiation of MC3T3-E1 Cells into Osteoblasts

**Figure 3.**
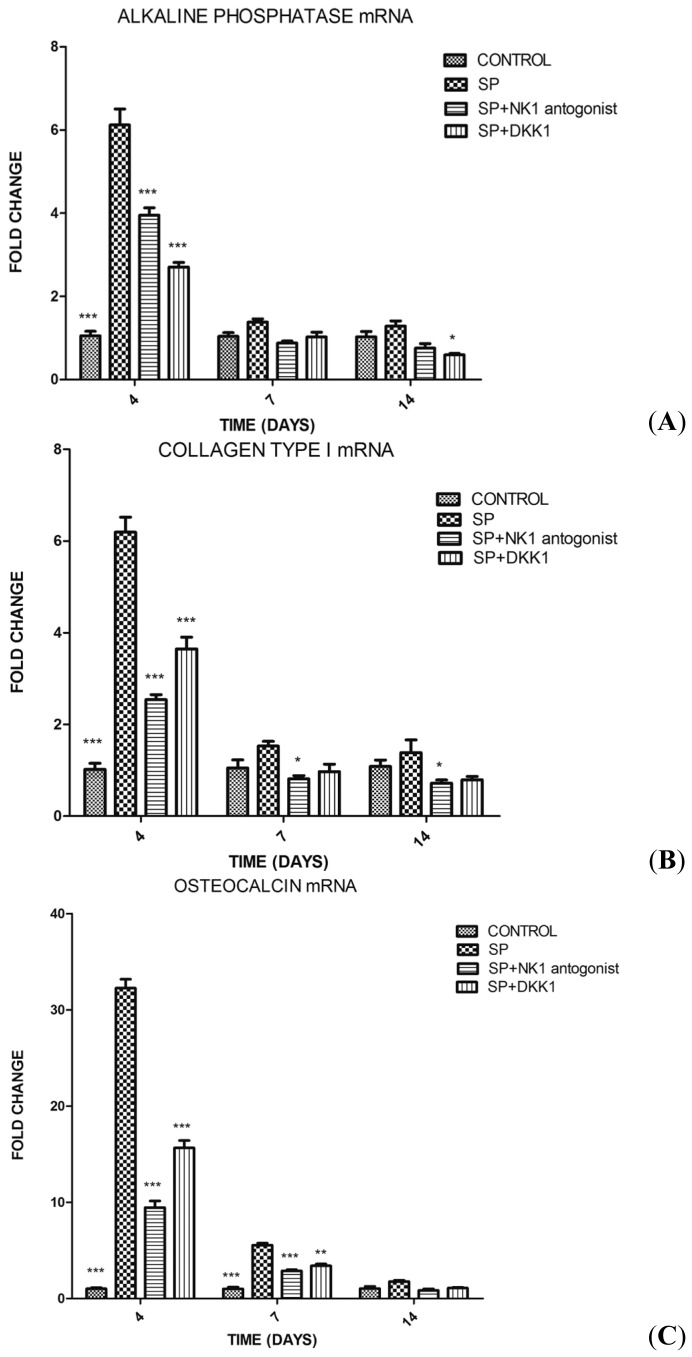

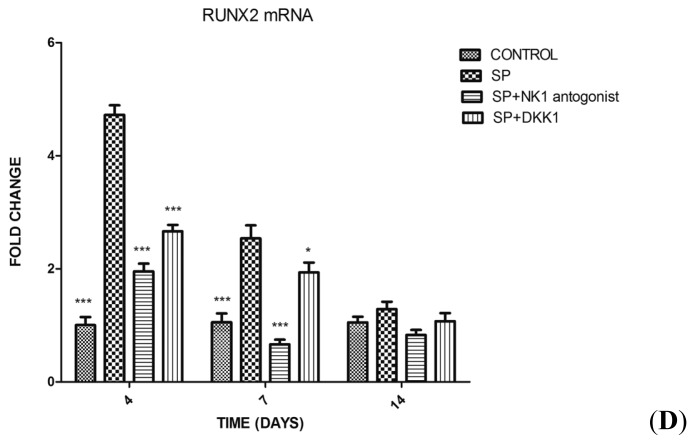
Effects of SP, SP + NK1 (neurokinin-1) antagonist, SP + DKK1 (Dickkopf-1) and the same amount of phosphate-buffered saline (PBS) on days 4, 7, 14 on gene expressions in MC3T3-E1 cells measured by quantitative polymerase chain reaction (qPCR). (**A**) alkaline phosphatase; (**B**) collagen type I; (**C**) osteocalcin; and (**D**) *Runx2* mRNA. Results are representative of 3 independent experiments. * *p* < 0.05 *versus* SP; ** *p* < 0.01 *versus* SP; and *** *p* < 0.001 *versus* SP, respectively.

qPCR revealed that SP (10^−8^ M) significantly increased the expression of alkaline phosphatase and collagen type I mRNA at day 4 compared with the control group (group D) and also significantly increased the levels of osteocalcin and *Runx2* mRNA on days 4 and 7 (Figure 3). Pretreatment of the cells with NK1 antagonist and DKK1 significantly inhibited the elevated expression of those genes upon SP treatment (Figure 3).

#### 2.1.4. Impact of SP, NK1 Antagonist and DKK1 on the Expression of Genes of the Wnt/β-Catenin Signaling Pathway

**Figure 4.**
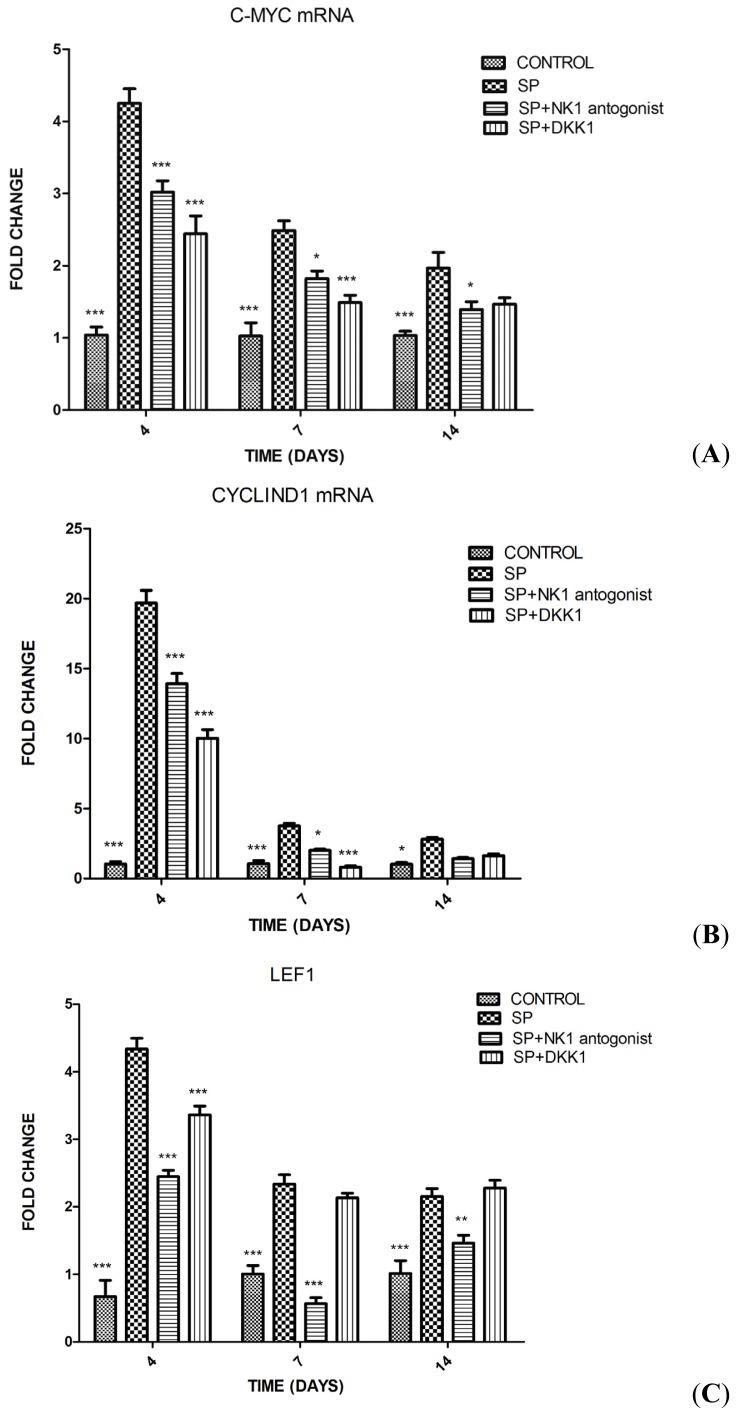

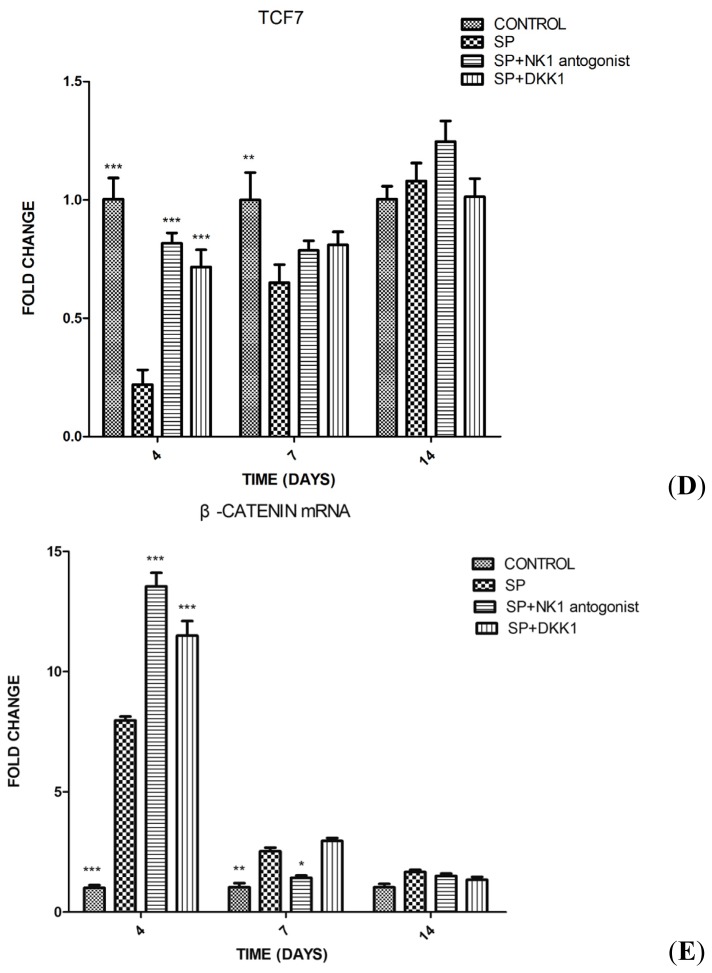
Effects of SP, SP + NK1 antagonist, SP + DKK1 and the same amount of PBS on days 4, 7, 14 on gene expressions in MC3T3-E1 cells measured by qPCR. (**A**) *c-myc* mRNA; (**B**) *cyclin D1* mRNA; (**C**) *Lef1*; (**D**) *Tcf7*; and (**E**) *β-catenin* mRNA. Results are representative of 3 independent experiments. * *p* < 0.05 *versus* SP; **p < 0.01 *versus* SP; and *** *p* < 0.001 *versus* SP, respectively.

To examine the potential mechanism of SP-induced osteoblast differentiation in MC3T3-E1 cells, gene expression of Wnt/β-catenin signaling pathway (*c-myc*, *cyclin D1*, *Lef1*, *Tcf7* and *β-catenin*) was evaluated by qPCR. The addition of SP (10^−8^ M) to the osteoinductive medium significantly increased the expression of *c-myc* and *Lef1* on days 4, 7, and 14 compared with the control group (group D). Moreover, it significantly increased the expression of cyclin D1 on days 4 and 7 but decreased the expression of *Tcf7* on days 4 and 7. These effects of SP were significantly inhibited by the pretreatment with NK1 antagonist and DKK1. SP also significantly increased the expression of β-catenin mRNA on day 4 but, instead of down-regulating the expression of β-catenin mRNA, the pretreatment with NK1 antagonist and DKK1 increased the expression of β-catenin mRNA compared with the SP group (group A) (Figure 4).

#### 2.1.5. Impact of SP, NK1 Antagonist and DKK1 on the Protein Levels of the Wnt/β-Catenin Signaling Pathway

**Figure 5.**
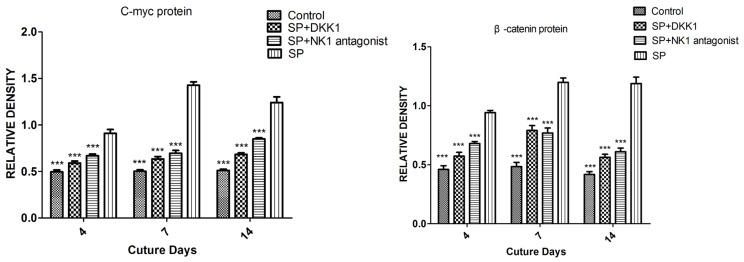

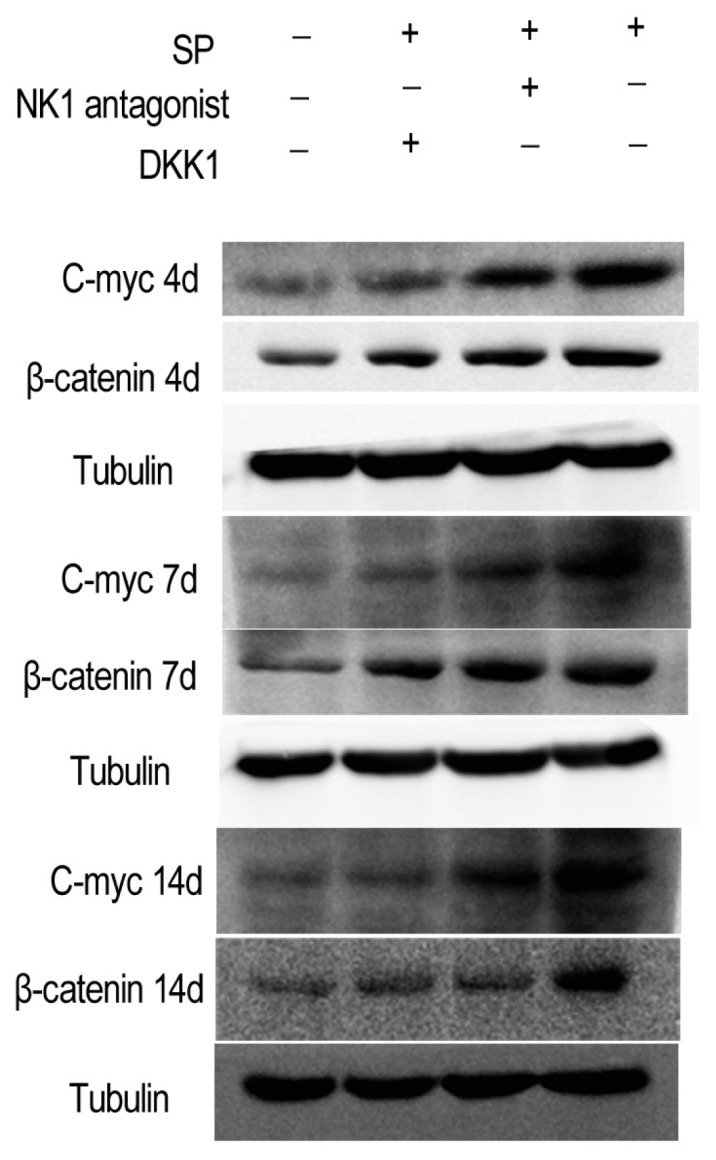
Effects of SP, SP + NK1 antagonist, SP + DKK1 and the same amount of PBS on days 4, 7, and 14 on β-catenin protein and c-myc protein expressions in MC3T3-E1 cells measured by Western blot analysis. *** *p* < 0.001 *versus* SP.

The addition of SP (10^−8^ M) to the osteoinductive medium increased the level of c-myc and β-catenin protein on days 4, 7, and 14 compared with the control group (group D). Pretreatment with NK1 antagonist and DKK1 inhibited the SP induced c-myc and β-catenin protein level increase on days 4, 7, and 14 (Figure 5).

#### 2.1.6. Impact of SP, NK1 Antagonist and DKK1 on β-Catenin Activation in MC3T3-E1 Cells

**Figure 6.**
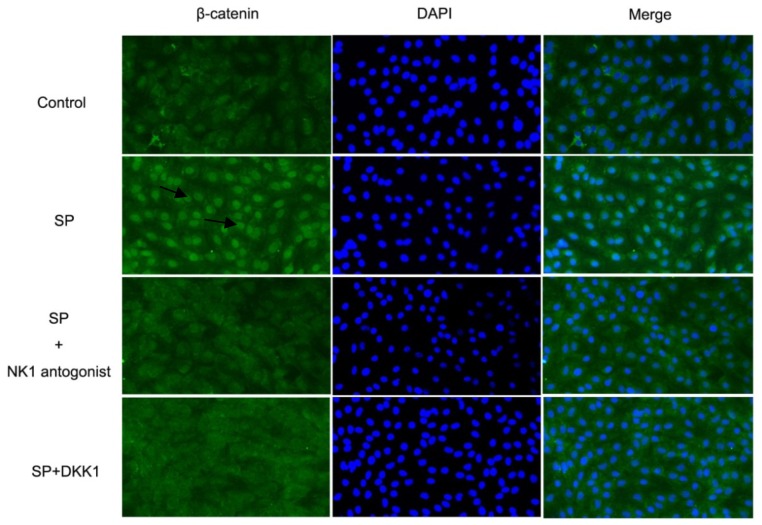
Effects of SP, SP + NK1 antagonist, and SP +DKK1 on β-catenin activation in MC3T3-E1 cells. Immunofluorescence staining with antibodies of β-catenin. Cells were incubated in the presence of SP, SP + NK1 antagonist, SP + DKK1, and the same amount of PBS for 4 days, then the β-catenin was detected by the immunostaining method as shown in green fluorescence (arrowheads). The nuclei were stained with 4′-6-diamidino-2-phenylindole (DAPI) as shown in blue.

We next examined whether the effect of SP on the activation of Wnt/β-catenin signaling was associated with an increase in β-catenin translocation to the nucleus. Immunolabeling and fluorescence microscopy of β-catenin in revealed low level of β-catenin in the nucleus of the MC3T3E1 cells from the control group (group D). Addition of SP (10^−8^ M) to the osteoinductive medium resulted in transfer of β-catenin (green) into the nucleus (blue). Pretreatment with NK1 antagonist and DKK1 prevented the SP induced transfer of β-catenin into the nucleus (Figure 6).

### 2.2. Discussion

In this study we explored the osteogenic effects of SP treatment on MC3T3-E1 osteoblast-like cells. We found that SP significantly promoted the differentiation of MC3T3-E1 cells. The osteoblastic differentiation *in vitro* is marked by expressions of osteoblastic genes, including *collagen type I*, *alkaline phosphatase*, *osteocalcin*, and *Runx2*. The role of SP in osteoblastic differentiation was investigated by detection of those four genes expression. Treatment of MC3T3-E1 cells with SP (10^−9^–10^−8^ M) led to an increase in osteoblastic gene expression including *collagen type I*, *alkaline phosphatase*, *Runx2*, and *osteocalcin* on day 4 and to a less extent on day 7. The role of SP was in turn inhibited by the NK1 receptor antagonist and DKK1, the antagonist of Wnt/β-catenin signaling pathway. SP treatment significantly increased the expressions of genes and levels of proteins associated with activation of the Wnt/β-catenin signaling pathway, but down-regulated the genes associated with inhibition of the Wnt/β-catenin signaling pathway on days 4, 7, and 14. The NK1 receptor antagonist and DKK1 inhibited these effects of SP. These findings indicate that SP may stimulate the differentiation of MC3T3-E1 cells via the Wnt/β-catenin signaling, whereas the NK1 antagonist and DKK1 may weaken the role of SP inducing the differentiation of MC3T3-E1 cells. Our data suggests a potential mechanism of nerves involvement in bone repair.

Previous studies have established the role of β-catenin as a key element of Wnt/β-catenin signaling pathway in control of developmental gene expression programs. Upon activation of the destruction complex, phosphorylation of β-catenin by GSK-3 triggers subsequent ubiquitination and degradation of β-catenin by the proteasome. This mechanism allows maintaining low level of β-catenin in the cytoplasm [15,19]. When the Wnt/β-catenin signaling pathway is activated, the destruction complex is inhibited, β-catenin protein accumulates in the cytoplasm and enters the nucleus where it interacts with Tcf/Lef transcription factors and contributes to activation of the expression of target genes [25–28]. In this study we examined the gene and protein expressions of β-catenin after the pretreatment of MC3T3-E1 cells with SP, NK1 antagonist + SP, and DKK1 + SP. We found that SP significantly increased the gene and protein expressions of β-catenin while NK1 receptor antagonist and DKK1 inhibited the expression of β-catenin protein, but increased the expression of β-catenin mRNA, so we speculate that the amount of β-catenin protein in the cytoplasm may depend mainly on the state of the destruction complex, but not mainly on the expression of β-catenin mRNA. As transferring of β-catenin into the nuclei is one of the most important steps in the activation of Wnt/β-catenin signaling pathway, we investigated the role of SP in promoting the transferring of β-catenin into the nuclei. We observed that SP promoted the accumulation and travelling of β-catenin to the nuclei. This may allow subsequent complex formation with Tcf/Lef and activation of Wnt target gene expression.

The role of Tcf7/Lef1 in the Wnt/β-catenin signaling is still controversial. Tcf7 may act as both a repressor and an activator while Lef1 might be usually an activator but sometimes a repressor [29–31]. In the dorsal midbrain, Lef1 and Tcf7 were shown to cooperatively activate the expression of the Wnt/β-catenin signaling target genes and promote cell proliferation [32]. In line with previous studies, we found that treatment of MC3T3-E1 cells with SP (10^−8^ M) for 4, 7, and 14 days led to increased expression of Lef1 and decreased expression of Tcf7. Therefore, we speculate that the cooperation of Lef1 and Tcf7 may be required in the process of activation of Wnt/β-catenin signaling induced by SP.

As Wnt/β-catenin signaling plays an important role in proliferation and differentiation in numerous developmental stages as well as adult tissue homeostasis, Wnt target genes have to be context specific [33,34]. In this study we investigated the expressions of two Wnt target genes and one corresponding protein (c-myc mRNA, cyclin D1 mRNA, and c-myc protein). Our results showed that SP remarkably increased the expressions of the two Wnt target genes and the one protein in MC3T3-E1 cells, indicating that the targeted genes, c-myc and cyclin D1, may be involved in the process of differentiation of MC3T3-E1 cells, which is promoted by the activation of the Wnt/β-catenin signaling pathway [35,36].

## 3. Experimental Section

### 3.1. Cell Culture

Murine osteoblastic MC3T3-E1 cells (ATCC, Manassas, VA, USA) were cultured in 5% CO_2_ atmosphere at 37 °C in α-MEM (minimum essential medium) containing 10% heat-inactivated fetal bovine serum (FBS). The medium was supplemented with 100 U/mL penicillin and 100 μg/mL streptomycin. After reaching 80% confluence, cells were passaged with 0.02% trypsin (Gibco, Grand Island, NY, USA) and transferred to new culture flasks in a ratio of 1:3. To induce osteogenic differentiation culture medium was changed to differentiation medium (α-MEM containing 10 mM β-glycerophosphate and 50 μg/mL ascorbic acid). The cell culture was performed in two steps. To find optimal concentration for promotion of cellular differentiation, the cells were divided into 4 groups according to different concentrations of SP (Sigma, S6883, St. Louis, MO, USA): Group 1, incubated with SP (10^−8^ M); Group 2, incubated with SP (10^−9^ M); Group 3 incubated with SP (10^−10^ M); and group 4, the control group, treated with the same volume of PBS. To determine the effects of SP, NK1 antagonist, and DKK1 on the differentiation of MC3T3-E1 cells, cells were divided into 4 groups: Group A, incubated with SP (10^−8^ M); Group B, incubated with a mixture of SP and NK1 antagonist (1 μM, Sigma, CP-96345) [37,38]; Group C, incubated with a mixture of SP and 0.2 μg/mL DKK1 (recombinant Human DKK-1, Peprotech, Rocky Hill, CT, USA) [39]; and Group D, the control group, added with the same amount of PBS.

### 3.2. Alizarin Red Staining and Alkaline Phosphatase Staining

Medium from the cultures before the shifting to differentiation medium was discarded and the cells were fixed in 4% paraformaldehyde for 30 min. After washing three times with ice-cold PBS, cells were stained for 5 min with alizarin red (Sigma) and analyzed under a light microscope.

The alkaline phosphatase (ALP) activity staining was performed with the cells before exposure to the differentiation medium. Cells were rinsed once with PBS, fixed in 2% formaldehyde and subsequently stained at 37 °C in solution containing naphthol AS-MX phosphate disodium salt, fast red salt (Sigma) and *N*,*N*-dimethyl formamide for 30 min or until a yellow color appeared. Cells were washed with PBS and photographed using a phase-contrast microscope. The alkaline phosphatase activity was assessed as red stains indicating the products of enzyme activity [40].

### 3.3. Quantitative Polymerase Chain Reaction (qPCR)

Total RNA of the MC3T3-E1 cells from the eight groups (groups 1–4 and groups A–D) at the 3 time points (4, 7, and 14 days) was extracted using a RNeasy Mini Kit (Qiagen, Valencia, CA, USA) according to the manufacturers protocol. After subsequent DNase digestion, 1 μg of RNA was used to synthetize 20 μL of cDNA using an iScript cDNA Synthesis Kit (Bio-Rad Laboratories, Hercules, CA, USA). Quantitative real time PCR reactions were performed in the ABI prism 7900 real time PCR system using the SYBR Green PCR master mix (Applied Biosystems, Foster City, CA, USA). Expression in each sample was evaluated in three technical replicates. Sequences of the oligonucleotides used in this study are listed in Table 1. Specificity of primer pairs and absence of primer dimmers was validated by analysis of the dissociation curves and the agarose gel electrophoresis. Comparative CT method was used for data analysis. Expression in each sample was normalized to the amount of glyceraldehyde 3-phosphate dehydrogenase (*GAPDH*) mRNA. Data represent mean expression from 3 experiments.

### 3.4. Protein Extraction and Western Blot

The cells from groups A–D at indicated time points (4, 7, and 14 days) were used for protein extraction. Cells were treated with the lysis buffer (Cell Signaling Technology, Danvers, MA, USA) and protein extracts were dissolved in sample buffer containing 50 mM Tris-HCl, 2% SDS, 10% glycerol, 100 mM dithiothreitol (pH = 6.80). Proteins were separated by SDS-PAGE in 10% polyacrylamide gel and transferred to a nitrocellulose membrane. Blots were decorated with anti-β-catenin antibodies and anti-c-myc antibodies (Santa Cruz Biotechnology, Santa Cruz, CA, USA). Bands were captured and documented through a CCD system (Imagestation 2000 MM, Kodak, Rochester, NY, USA). Blots were stripped and re-probed with anti-tubulin antibodies to demonstrate equal loading and to allow normalization of the protein content. Densitometry of the bands was performed using Molecular Imaging Software Version 4.0 (Kodak).

### 3.5. Immunofluorescence Staining

The cells from groups A–D at day 4 were fixed (4% paraformaldeyde in PBS, 15 min, room temperature), permeabilized (0.25% TritonX100 in PBS, 15 min) and blocked in 1% bovine serum albumin (BSA) in poly butylene succinate-*co*-butylene terephthalate (PBST) for 30 min. After washing three times in PBS, cells were incubated overnight with rabbit anti-mouse anti-β-catenin antibodies (Santa Cruz Biotechnology) diluted 1:100 in PBST. Cells were washed 3 times with PBS and incubated for 1 h with fluorescein isothiocyanate (FITC)-linked rabbit anti-mouse IgG antibody diluted 1:100 (USCN) and again washed three times in PBS. 4′-6-Diamidino-2-phenylindole (DAPI) was added to mark the nuclei. Slides were examined in Fluoview 300 (Olympus, Tokyo, Japan) fluorescent microscope and images were acquired [4,41].

### 3.6. Statistical Analysis

Statistical analyses were performed in SPSS (version 13.0, IBM, Armonk, NY, USA). One-way ANOVA test followed by Dunnett (2-tailed t) *post hoc* test was used to evaluate the statistical significance of the differences. Significance was achieved when *p* < 0.05. Presented error bars indicate standard error of the mean (SEM). * is indicative of *p* < 0.05, ** *p* < 0.01 and *** *p* < 0.001.

## 4. Conclusions

In conclusion, this study demonstrates that SP may stimulate the osteogenic differentiation of MC3T3-E1 cells and the canonical Wnt signaling may contribute to this process. The role of SP and Wnt/β-catenin signaling in osteogenesis should be further and depth studied by detecting more genes and proteins, such as GSK-3β, Axin, and APC, to elucidate the role of SP and Wnt/β-catenin signaling in bone development and remodeling to improve our understanding of the role of nerves in bone repair.

## Figures and Tables

**Table 1.** Sequences of the primers used in this study.

Gene	Source	Sequence	Predicted length (bp)
*ALP*	NM_007431	5′-AACCCAGACACAAGCATTCC-3′	200
		5′-GCCTTTGAGGTTTTTGGTCA-3′	
*COL1*	NM_007742	5′-GCCAAGAAGACATCCCTGAA-3′	107
		5′-GCCATTGTGGCAGATACAGA-3′	
*OCN*	NM_007541	5′-TGACAAAGCCTTCATGTCCA-3′	175
		5′-TTTGTAGGCGGTCTTCAAGC-3′	
*RUNX2*	NM_009820	5′-AAGTGCGGTGCAAACTTTCT-3′	175
		5′-ACGCCATAGTCCCTCCTTTT-3′	
*C-MYC*	NM_001177353	5′-TCCTGTACCTCGTCCGATTC-3′	195
		5′-GGTTTGCCTCTTCTCCACAG-3′	
*CCND1*	NM_007631	5′-GCGTACCCTGACACCAATCT-3′	183
		5′-CTCCTCTTCGCACTTCTGCT-3′	
*LEF1*	NM_010703	5′-TATGAACAGCGACCCGTACA-3′	132
		5′-TCGTCGCTGTAGGTGATGAG-3′	
*TCF7*	NM_009331	5′-ATCCTTGATGCTGGGATCTG-3′	139
		5′-CTTCTCTGCCTTGGGTTCTG-3′	
*β-CATENIN*	NM_007614	5′-ATGGCTTGGAATGAGACTGC-3′	150
		5′-ATGCTCCATCATAGGGTCCA-3′	
*GAPDH*	NM_008084	5′-ATTGTCAGCAATGCATCCTG-3′	102
		5′-ATGGACTGTGGTCATGAGCC-3′	
